# Assessing the impact of integrated community case management (iCCM) programs on child mortality: Review of early results and lessons learned in sub–Saharan Africa

**DOI:** 10.7189/jogh.04.020411

**Published:** 2014-12

**Authors:** Agbessi Amouzou, Saul Morris, Lawrence H Moulton, David Mukanga

**Affiliations:** 1 Data and Analytics, UNICEF, New York, NY, USA; 2 Children’s Investment Fund Foundation, London, UK; 3 Department of International Health, Johns Hopkins Bloomberg School of Public Health, Baltimore, MD, USA; 4 Bill & Melinda Gates Foundation, Seattle, WA, USA

## Abstract

**Aim:**

To accelerate progress in reducing child mortality, many countries in sub–Saharan Africa have adopted and scaled–up integrated community case management (iCCM) programs targeting the three major infectious killers of children under–five. The programs train lay community health workers to assess, classify and treat uncomplicated cases of pneumonia with antibiotics, malaria with antimalarial drugs and diarrhea with Oral Rehydration Salts (ORS) and zinc. Although management of these conditions with the respective appropriate drugs has proven efficacious in randomized trials, the effectiveness of large iCCM scale–up programs in reducing child mortality is yet to be demonstrated. This paper reviews recent experience in documenting and attributing changes in under–five mortality to the specific interventions of a variety of iCCM programs.

**Methods:**

Eight recent studies have been identified and assessed in terms of design, mortality measurement and results. Impact of the iCCM program on mortality among children age 2–59 months was assessed through a difference in differences approach using random effect Poisson regression.

**Results:**

Designs used by these studies include cluster randomized trials, randomized stepped–wedge and quasi–experimental trials. Child mortality is measured through demographic surveillance or household survey with full birth history conducted at the end of program implementation. Six of the eight studies showed a higher decline in mortality among children 2–59 months in program areas compared to comparison areas, although this acceleration was statistically significant in only one study with a decline of 76% larger in intervention than in comparison areas.

**Conclusion:**

Studies that evaluate large scale iCCM programs and include assessment of mortality impact must ensure an appropriate design. This includes required sample sizes and sufficient number of program and comparison districts that allow adequate inference and attribution of impact. In addition, large–scale program utilization, and a significant increase in coverage of care seeking and treatment of targeted childhood illnesses are preconditions to measurable mortality impact. Those issues need to be addressed before large investments in assessing changes in child mortality is undertaken, or the results of mortality impact evaluation will most likely be inconclusive.

The fourth Millennium Development Goal (MDG4) of reducing child mortality by two–thirds provided an impetus to countries to develop and scale–up focused strategies and programs to accelerate reduction in child mortality with increased international support [1]. However, in the mid–2000s, slow progress was observed in many countries, especially in sub–Saharan Africa where child mortality is highest. This meant that focus must not only be directed at scaling up high impact life–saving interventions targeting main killers of children, but also on the monitoring and evaluation of these programs [1–3]. Integrated community case management (iCCM) is one such approach that is expected to produce immediate impact on mortality if implemented in optimal conditions, given that it directly tackles the key proximate determinants of child survival. It increases access of children to prompt and effective care and treatment for the three main killer infections using effective interventions, while also managing acute malnutrition. Therefore, if high quality iCCM is properly delivered by well–trained community health workers, targeting children who are most in need (those with limited or no access to care), and if the program is utilized by a large portion of the target population, accelerated reduction in child mortality should be expected [4,5]. This impact model is theoretically plausible and appealing, especially given that each individual intervention included in iCCM has already been proven efficacious in controlled trials.

Countries in sub–Saharan Africa, where the burden of child death is the largest, were therefore encouraged to adopt and scale–up iCCM, focusing on pneumonia, diarrhea and malaria, and in many cases also malnutrition [6]. However, there is currently no demonstrated impact of large scale iCCM on child survival in Africa. The success of efficacy trials of individual interventions does not always translate into effectiveness of either the individual interventions or integrated programs. This can be explained by health system constraints and demand side barriers that are often faced in large scale “real–life” programs.

Furthermore, previous impact evaluation studies of CCM were mainly conducted in Asian contexts, focusing in general on a single or two diseases [7]. Three meta–analyses of CCM of pneumonia conducted between 1992 and 2010 included only one study from a country in sub–Saharan Africa, among the 15 countries reviewed [8–10]. More recent studies have demonstrated the feasibility of CCM for a single disease (or two diseases) in Africa and usefulness of using community health workers for the provision of CCM [11–15].

A recent review of the evidence of the effectiveness of CCM in reducing pneumonia burden suggested the lack of evidence in Africa and poor adherence of community health workers to the guidelines [16]. The review focused mostly on pneumonia and did not include several other studies that showed feasibility of the use of community health workers (CHWs) and acceptable quality of care provided by CHWs in comparison to care provided at first level health facilities [17,18]. One could view iCCM of childhood illnesses as an adaptation of the integrated management of childhood illnesses (IMCI) program at community level with focus on community health worker. In 2010, UNICEF and WHO endorsed the iCCM program as a key strategy for reaching larger populations with effective care and reducing inequity [4].

However, the implementation of IMCI has not always been associated with demonstrable changes in child mortality [19,20]. The multi–country evaluation of the IMCI in Bangladesh, Brazil, Peru, Tanzania, and Uganda used various designs across these countries but showed positive impact of child mortality in Tanzania only [21]. In Bangladesh, where the evaluation used the strongest design, based on a cluster randomized trial with health facility catchment areas randomized to intervention and comparison areas, no statistically significant effect was found on child mortality. This absence of demonstrable effect stands in contrast to positive effects at health facility level in terms of health worker skills and health system support, and at community level in terms of family and community practices [22].

Therefore, the large support and investment in the scale–up of iCCM in Africa was under some pressure to demonstrate mortality impact of the program within a short time period. Countries and implementing partners faced a dual challenge. They needed to ensure the implementation of high quality large scale iCCM programs, targeting mothers and children that had hitherto poor or no access to health care. They also needed to be open to an evaluation design that allowed rigorous assessment of the impact of the program on child mortality. The evaluation of a large scale “real–life” program such as iCCM comes with added challenges that must be borne in mind at the design stage, especially when a mortality impact assessment component is included. The opportunities for a randomized design are rare, and even quasi–experimental designs are becoming increasingly difficult to implement, given the difficulty in identifying adequate comparison areas. In the rare cases where it is possible to randomize, there is also a risk to external validity as the evaluation may become so controlled and context specific that its generalizability can be questioned [20,23,24].

In 2013, the Bill & Melinda Gates Foundation (BMGF) and UNICEF launched an initiative to conduct a comprehensive review of the evidence in support of iCCM in Sub–Saharan Africa, take stock of the experience and lessons learned in terms of program implementation and evaluation. As a part of this initiative, we reviewed recent studies or program evaluations that incorporated an assessment of mortality impact of iCCM, whether already published or unpublished. We assessed the strength of the evidence in these studies by rigorously looking at how the mortality component of the study was designed and implemented within the overall evaluation design. We provide a summary of lessons learned and recommendation for iCCM mortality impact evaluation designs in the future.

## METHODS

We started the review by searching the literature for studies that assessed the mortality impact of integrated community case management programs. We searched PubMed, EMBASE, BIOSIS, Web of Science and Cochrane library to identify relevant studies reported in English, and published at any time until September 2013. The subject headings are listed in **Box 1**.

Box 1 **Subject headings for systematic search of the studies that evaluated the impact of the ICCM programs on child mortality**

Community Health Aide and Mortality; Community Health Worker and Mortality; Village Health Worker; Volunteer Health Worker; Malaria Pneumonia Diarrhea Integrated; malaria and pneumonia and diarrhea and impact; Village Health Volunteer; Home Management of Fever and Impact; Community Health Volunteer; Lay Health Worker; Community Case Management and Mortality; and, integrated community case management and mortality.

This search did not identify any study that actually tested the impact of iCCM based on the three main diseases of interest. Only one study, conducted in Ghana, tested the management of pneumonia and malaria vs malaria alone, using a comparison area with no iCCM and cluster randomized control design [25]. We then contacted Non-governmental organizations (NGOs) or academic institutions known to have conducted recent evaluations of iCCM programs that included child mortality assessment. Although these studies were not yet published, all data collection had been completed and most were at the data analysis stage or at the report writing stage. We used three criteria for retention of a study for review: (i) the evaluation design must include an intervention area where the iCCM is implemented and a comparison area, (ii) the evaluation must include rigorous assessment of mortality impact through primary data collection using either household survey with full birth or pregnancy history, or population surveillance; and (iii) the researchers must be willing to share their micro–data on mortality to allow data quality assessment of the data sets and reanalysis.

As criteria for mortality data quality, we excluded all studies in which the measured baseline mortality rate was substantially lower than the mortality of the entire rural population of the same country at the same time. We did not consider studies in which only a handful of providers had been trained, regarding them as unlikely to be informative about the impact of large–scale national or sub–national programs. Eventually, we identified a total of eleven recently completed iCCM impact evaluation studies. Of these studies, three were excluded for poor mortality data quality (South Sudan), different mortality assessment method (Sierra Leone/IRC), or unavailability of micro data sets (Uganda Eastern). Only the remaining eight studies are discussed in this paper. **Table 1** includes the list of the eight studies, along with the organization that carried out the evaluation and the study year.

**Table 1:** Evaluation studies included in the analysis

Country	Partner support	Study year	Study title
Burkina Faso	Groupe de Recherche Action en Santé and WHO/Tropical Disease Research (TDR)	2010–2013	Home and community management of fevers/malaria and pneumonia in children under–five: *a cluster randomised controlled trial of an integrated approach in a rural district of Burkina Faso*
Cameroon	Population Services International	2009–2012	Cameroon CCM Endline Evaluation 2012: outcomes and impact in Doumé and Nguelemendouka districts after three years of program implementation
Ethiopia	Johns Hopkins University	2011–2013	Independent evaluation of the Integrated Community Case Management of common childhood illnesses in Oromia region, Ethiopia
Ghana	Ghana Health Services and WHO/Tropical Disease Research (TDR)	2006–2009	Impact of Community Management of Fever (Using Antimalarials With or Without Antibiotics) on Childhood Mortality: A Cluster–Randomized Controlled Trial in Ghana
Sierra Leone	UNICEF	2010–2012	Health for the Poorest quintile – Sierra Leone
Uganda (Central)	UNICEF and Malaria Consortium	2010–2011	Health for Poorest Quintile Project – Uganda
Uganda (Western)	Malaria Consortium	2009–2012	Improving Access For Under–Fives To Life Saving Treatment Through Integrated Community Case Management For Malaria, Pneumonia And Diarrhoea – Uganda
Zambia	Malaria Consortium	2010–2012	Improving Access For Under–Fives To Life Saving Treatment Through Integrated Community Case Management For Malaria, Pneumonia And Diarrhoea – Zambia

Six of the eight studies included an endline mortality data based on household survey with full birth history module administered to women aged 15–49. Full birth history module consists of questions to women age 15–49 on all live births they ever had, the date of birth, and the survival status for each birth. For children who had died, age at death was also collected. The full birth history data has the advantage of allowing direct child mortality computation on retrospective periods up to 15–25 years preceding the survey, thus providing the possibility of measuring mortality on defined baseline and endline periods. We therefore used endline mortality data to compute mortality among children 2–59 months at baseline and endline, in intervention and comparison areas. The use of a single data set to measure child mortality at baseline and endline is very convenient. It avoids differential measurement errors that could have resulted from the use of two different data sets. The age group 2–59 months was used, because it is the main target of the iCCM program. The mortality measurement period at endline was determined from the time when at least 80% of the community health workers (CHWs) were trained in iCCM and deployed to provide services. This period was retained to ensure that mortality was assessed when the program was functioning at full scale and likely to be producing effect in the target population [20]. Once the endline measurement period was defined, we retrospectively defined a baseline period that was anterior to the program implementation and was identical in length and season. This was necessary to rule out any seasonality effect on the assessment of the mortality impact.

We used a cross–sectional random effects Poisson model to estimate the ratio of ratios in death occurrence among children aged 2–59 months between baseline and endline, and also across intervention and comparison areas. The ratio was estimated as the interaction coefficient between the time (endline vs baseline) and intervention (intervention area vs comparison area). The analysis adjusted for clustering at district level by introducing a district–level random intercept. Computations were conducted using STATA 12.0.

## RESULTS

### Overall design of mortality studies

The eight recent studies reviewed cover West and Central Africa (Burkina Faso, Cameroon, Ghana and Sierra Leone) and East Africa (Ethiopia, Uganda and Zambia) and thus represent a variety of African contexts (**Tables 1**** and ****2**). They were all conducted at subnational level, ranging from a few to many districts, and do not represent evaluations of their entire national iCCM scale–up programs. Although all the studies were conducted to determine the effectiveness of iCCM in reducing child mortality, some countries such as Ethiopia have already moved to full national scale–up based on recommendations from WHO and UNICEF [4]. Three of the studies (Burkina Faso, Ethiopia and Ghana) used randomized controlled trial design and the remaining used quasi–experimental design with non–random selection of intervention and comparison areas. The three studies that used the strongest evaluation design have some particularities worth noting. The Burkina Faso and Ghana studies were designed and carried out in collaboration with WHO/TDR in a district each, covering populations of respectively 380 000 and 110 000 individuals. Villages or groups of communities served as clusters and were randomized to intervention and control areas. In addition, the Ghana study included only CCM of fever, while the Burkina Faso study included CCM of fever and pneumonia. Both studies used a randomized stepped–wedge approach and ran for approximately three years. Further details of the approach are described elsewhere [25]. To ensure analytic comparability to other studies reviewed, the analysis of data from the Ghana study was restricted to the period when fever was managed with an antimalarial drug in combination with antibiotics. In Burkina Faso, the analysis was restricted to the period when CCM of both fever and pneumonia was implemented in the intervention area, while the control area received no CCM. The Ethiopia study was conducted by researchers from the Johns Hopkins University in two zones covering 31 districts and a population larger than 4.2 million. All 31 districts were randomly assigned to intervention and comparison areas. Although the intervention areas received the enhanced iCCM program, which was initiated in Ethiopia in 2010 and included CCM of all three illnesses (malaria, pneumonia and diarrhea), the comparison areas received the existing CCM of malaria and diarrhea. Thus, in theory, the main difference between the intervention and the comparison areas was the introduction of CCM for pneumonia in the intervention area. However, the iCCM program in Ethiopia had been completely redesigned, with five day refresher training of the community health workers, continuous provision of drugs and commodities, and improved monitoring and supervision. Details about the Ethiopia study are provided elsewhere [18].

**Table 2:** Characteristics of the design of iCCM) evaluation studies

Country	Study design	Number of intervention districts/clusters	Number of comparison districts/clusters	Type of CHWs providing iCCM	Method for mortality measurement	Sample size for the endline mortality survey (No. HHs)	Mortality measurement period	Difference in differences estimate of mortality rate ratio among children age 2–59 mo and 95%CI
Burkina Faso	RCT	19 × 19*	19*	Volunteers	DSS	76 000‡	11 mo	0.95 (0.57–1.59)
Cameroon	Quasi–experimental	2	1	Volunteers	Census with FBH	18 177	35 mo	1.05 (0.85–1.29)
Ethiopia	RCT	16	15	Paid Government CHW	Survey with FBH	28 000	18 mo	0.85 (0.62–1.18)
Ghana	RCT	39 × 37^†^	38^†^	Volunteers	DSS	22 000‡	11 mo	0.24 (0.06–0.96)
Sierra Leone	Quasi–experimental	2	2	Volunteers	Survey with FBH	6 000	18 mo	0.79 (0.41–1.51)
Uganda (Central)	Quasi–experimental	8	3	Volunteers	Survey with FBH	8 000	11 mo	0.70 (0.18–2.78)
Uganda (Western)	Quasi–experimental	9	3	Volunteers	Survey with FBH	8 000	22 mo	0.66 (0.32–1.40)
Zambia	Quasi–experimental	4	3	Volunteers	Survey with FBH	8 000	16 mo	1.45 (0.86–2.46)

^i^CCM – integrated community case management, CHW – community health worker, HH – households, FBH – full birth history, DSS – Demographic Surveillance Systems, mo – months

*in Burkina Faso, 57 clusters consisting of villages were randomized to three arms for a stepped wedge design: during the initial phase, 19 clusters were randomly allocated to intervention areas consisting of CCM of fever with antimalarial (arthemeter/lumefantrine) and pneumonia with antibiotics (Cotrimoxazole); 19 clusters were allocated to another intervention areas consisting of CCM of fever with antimalarial drug, and 19 clusters were allocated to control.

^†^In Ghana, 114 clusters consisting of group of communities were randomized to three arms for a stepped wedge design: during the initial phase 39 clusters to intervention consisting of CCM of fever with an antimalarial (Artesunate Amodiaquine) plus an antibiotic (amoxicillin), 37 clusters to intervention consisting of CCM of fever with antimalarial only (Artesunate Amodiaquine), and 38 clusters served as control.

‡Represents an estimate of the total number of households in the district where the demographic surveillance system is implemented. It was determined by dividing the total population by an estimated average household size of 5.

The remaining five studies used a quasi–experimental design, with only a few districts where intervention was implemented and a few districts for comparison. In general, the number of intervention districts was higher than the number of comparison districts.

Sample sizes varied tremendously across the studies. Of the studies that used household surveys with full birth history for mortality assessment, the study in Ethiopia had the largest sample size (28 000 households). The smallest sample size was assembled in Sierra Leone. The two studies conducted in DSS sites covered the entire population of the district.

### Mortality measurement

The Burkina Faso and Ghana studies were conducted in a district with on–going demographic surveillance system and therefore relied on the surveillance approach for mortality assessment. In both countries, a biannual census of the entire study district was conducted, complemented with continuous monitoring of births and deaths by key informants. While surveillance of births and deaths in communities generally suffers from under–reporting and leads to child mortality rates that can be grossly underestimated, complementing the approach with biannual census of the target population helps to improve completeness [26,27]. However, unless a well–functioning DSS is in place, it is impractical to rely on vital events surveillance for mortality assessment in most African countries. All other six studies have therefore relied on full birth histories for child mortality assessment. **Table 2** presents the length of the mortality measurement period for each study. Across all eight studies, this period ranges from 11 months in the Burkina Faso, Ghana and Central Uganda studies to 35 months in the Cameroon study. Except in Cameroon, this period is under two–years for all studies and under one year for three studies (Burkina Faso, Ghana, and Uganda Central). It should be noted that for Burkina Faso and Ghana the period represents only the first phase in the stepped wedge design and does not represent the entire duration of the implementation scheme.

**Table 2** also presents the estimate of the mortality rate ratios among children age 2–59 months between intervention and comparison areas. Six of the eight studies show a ratio below 1 suggesting consistently larger mortality decline among children 2–59 months in intervention compared to comparison areas. However, this acceleration is statistically significant only in the study in Ghana, where there was an excess decline of 76% in intervention compared to comparison areas. Interestingly, in Cameroon and Zambia mortality among children 2–59 months appears to have declined much more slowly in intervention areas than in comparison areas.

## DISCUSSION

Although iCCM programs are being scaled up in many African nations, the effectiveness of the strategy in accelerating decline in mortality among children under–five is yet to be fully demonstrated. We reviewed recent studies that attempted to measure the mortality impact of iCCM in the African context. At total of eight studies were identified and included in the current review. Six of these studies assessed real–life iCCM programs that included the management of the three high–burden illnesses – pneumonia, malaria and diarrhea. Two studies were implemented and carried out in a demographic surveillance system site, assessing the effectiveness of CCM of fever (and pneumonia) with antimalarial drugs and antibiotics. The eight studies used different evaluation designs, including randomized cluster designs, randomized stepped–wedge designs and quasi–experimental designs. The large heterogeneity in the programs being evaluated and the evaluations design precluded a meta–analysis of the mortality results. However, six of the eight studies showed greater decline in mortality among children 2–59 months in intervention areas compared to comparison areas, although this acceleration was statistically significant in only one study.

This review demonstrated that some strategies have worked well in evaluating the mortality impact of iCCM programs. First, the collection of mortality data using full birth histories is a promising approach for the evaluation of iCCM programs.

Birth history data, collected at a single moment in time towards the end of the program implementation period, permits the reconstruction of the evolution of mortality in the target population over at least the previous two decades, with the possibility of zooming in on specific periods. The ability to understand mortality trends before the introduction of the iCCM program aids the interpretation of the evaluation findings. The fact that both pre–implementation and implementation data are collected from the same households favours a valid statistical analysis. Second, in some of the studies, the intervention was introduced in a mosaic of small geographic areas, rather than in a few large areas such as districts. Those intervention ‘clusters’ were then compared to a similar number of non–program or ‘comparison’ areas. The strategy permitted relatively straightforward inference about the likely impact of the same intervention across a larger population. It also generally resulted in intervention and comparison groups starting at similar levels of pre–program mortality. Finally, some studies included the collection of a comprehensive data set including not only mortality but also changes in treatment coverage (for both iCCM and non–iCCM interventions) and detailed program utilization data. This greatly facilitated the interpretation of the mortality findings [28,29].

However, we noted several limitations in the impact evaluation studies. Collecting birth histories in minimally literate populations requires careful training of fieldworkers and intensive supervision of the data collection process, which was not achieved in all cases. Since it is difficult to detect poor quality mortality data after it has been collected, we relied entirely on an assessment of the plausibility of the levels of mortality assessed at baseline; likely, some moderately poor quality data passed this test, which lacks sensitivity. Two studies took advantage of existing Demographic Surveillance Systems (DSS) but these are special opportunities that are not readily available everywhere or in large areas.

There were basic flaws in the evaluation design of the majority of studies, making it very difficult to draw any inference from the results. Comparison areas were either different from the interventions at baseline, and/or the program was allocated to a very small number of relatively large geographic areas, making it impossible to rule out the influence of idiosyncratic local changes on the findings. It is also possible that the comparison areas received some form of CCM, as was the case in Ethiopia. In several cases, the program delivered in the intervention areas was so different from the standard model of iCCM that the value of comparing across studies has to be questioned. This is one of the main reasons why we avoided an attempt at meta–analysis of all 8 studies to establish an effectiveness of iCCM in sub–Saharan Africa in “real life” condition.

Because mortality is a rare event, virtually none of the studies was adequately powered to detect a statistically significant impact of the program following a short implementation period. Power calculations, which are a basic step in the development of an evaluation plan, were either simply not done, or were based on out–of–date or over–optimistic assumptions, or did not take the evaluation design into account. In addition, program exposure periods were either far too short to accumulate sufficient numbers of deaths in the study population, and/or did not give the targeted populations time to get used to using the new providers.

The programs took place in areas with very rapidly evolving health systems and epidemiological contexts. Thus, they often no longer met basic assumptions required to demonstrate mortality impact as described in the paragraph below.

These early iCCM mortality impact evaluation studies provide several lessons for future evaluations. iCCM programs intervene to directly prevent deaths from the most common life–threatening diseases in resource–constrained communities. As such, it might be assumed that iCCM programs will result in lower mortality rates. However, in order for this to be demonstrated, three sets of conditions must be met. First, the program must be delivered at an intensity sufficient to generate impact at a population level. The theory of change for an iCCM program indicates that, in order to generate mortality impact, there must be a substantial change in the proportion of sick children in the target population who receive safe, effective and timely treatment. In order for treatment coverage to increase, utilization of the new providers must be high and their service quality reliably adequate. Furthermore, the number of iCCM providers deployed must be sufficient to have substantially increased overall density of service delivery points. These basic preconditions have not always been met. Second, iCCM program design must be appropriate for the context, including treatment for all of the most important life–threatening conditions, medicines that are locally effective, effective targeting at children who are truly at risk of dying, and a relative scarcity of alternative providers. This set of assumptions has also not universally been met, with many programs continuing to use cotrimoxazole for the treatment of pneumonia, for example, in spite of ample evidence of bacterial resistance to cotrimoxazole. In addition, some studies have shown that substantial proportion of children with non–severe pneumonia may only have wheeze or non–bacterial pneumonia and do not require antibiotics treatment at all [30]. Third, the methods of assessing mortality impact must be reliable, precise and generalizable.

Mortality impacts in the studies reviewed vary considerably, from a (statistically significant) 76% reduction in mortality, to a (non–significant) 43% increase in mortality, with a median reduction of 21%. We believe that much of this apparent variation is due to inappropriate program and evaluation design. Mortality measurement is a very specialized activity requiring well–trained interviewers, close supervision of fieldwork, and — above all —very large survey sample sizes. Measuring directly mortality for impact assessment requires a large investment.

There is a logical chain of iCCM results in which high utilization of quality services is the precondition for high coverage of safe, effective, and timely treatment of sick children. The latter is, in turn, the precondition for observing reduced mortality. Mortality measurement should not be undertaken unless it can be demonstrated that the other preconditions have already been met. As a rule, mortality outcomes should probably not be considered for any programs likely to have been implemented for less than two years.

Because of rapidly changing health systems and epidemiological contexts, it is much easier to interpret mortality data if companion data on treatment coverage, program utilization, and other contextual variables were also collected. Those indicators are desirable for program monitoring in any case.

Program design considerations often conflict with the basic premises of good evaluation. For example, one program decided to introduce iCCM in two districts and compare their mortality experience with one comparison district. This “2 versus 1” comparison is known in the evaluation literature to produce results which are impossible to interpret. Likewise, pushing programs into the highest mortality districts inevitably means that comparison areas will not be truly comparable at baseline, creating extreme difficulties of interpretation of evaluation findings later on.

If a strong evaluation context can be guaranteed, full birth histories – or, better still, full *pregnancy* histories – are the ideal way of collecting data on child mortality. They should be analyzed by compartmentalizing both deaths and person–years at risk into multiple sequential time periods. A single birth history survey can produce information both for the program implementation period and for the pre–program period.

## References

[1] United Nations. The Millennium Development Goals Report. New York: UN, 2014.

[2] Jones G, Steketee RW, Black RE, Bhutta ZA, Morris SS, Bellagio Child Survival Study Group (2003). How many child deaths can we prevent this year?. Lancet.

[3] Bryce J, el Arifeen S, Pariyo G, Lanata CF, Gwatkin D, Habicht JP (2003). Reducing child mortality: can public health deliver?. Lancet.

[4] World Health Organization, United Nation Children’s Fund. Integrated community case management (iCCM). Geneva: WHO & UNICEF, 2012.

[5] Bryce J, Victora CG, Boerma T, Peters DF, Black RE (2011). Evaluating the scale–up for maternal and child survival: a common framework.. Int Health.

[6] Liu L, Johnson HL, Cousens S, Perin J, Scott S, Lawn JE (2012). Global, regional, and national causes of child mortality: an updated systematic analysis for 2010 with time trends since 2000.. Lancet.

[7] Bang AT, Bang RA, Tale O, Sontakke P, Solanki J, Wargantiwar R (1990). Reduction in pneumonia mortality and total childhood mortality by means of community–based intervention trial in Gadchiroli, India.. Lancet.

[8] Sazawal S, Black RE (1992). Meta–analysis of intervention trials on case management of pneumonia in community settings.. Lancet.

[9] Sazawal S, Black RE, Pneumonia Case Management Trials Group (2003). Effect of pneumonia case management on mortality in neonates, infants, and preschool children: a meta–analysis of community–based trials.. Lancet Infect Dis.

[10] Theodoratou E, Al–Jilaihawi S, Woodward F, Ferguson J, Jhass A, Balliet M (2010). The effect of case management on childhood pneumonia mortality in developing countries.. Int J Epidemiol.

[11] Marsh DR, Gilroy KE, Van de Weerdt R, Wansi E, Qazi S (2008). Community case management of pneumonia: at a tipping point?. Bull World Health Organ.

[12] Ajayi IO, Browne EN, Bateganya F, Yar D, Happi C, Falade CO (2008). Effectiveness of artemisinin–based combination therapy used in the context of home management of malaria: a report from three study sites in sub–Saharan Africa.. Malar J.

[13] Mukanga D, Tiono AB, Anyorigiya T, Kallander K, Konate AT, Oduro AR (2012). Integrated community case management of fever in children under five using rapid diagnostic tests and respiratory rate counting: a multi–country cluster randomized trial.. Am J Trop Med Hyg.

[14] Winch PJ, Gilroy KE, Wolfheim C, Starbuck ES, Young MW, Walker LD (2005). Intervention models for the management of children with signs of pneumonia or malaria by community health workers.. Health Policy Plan.

[15] Yeboah–Antwi K, Pilingana P, Macleod WB, Semrau K, Siazeele K, Kalesha P (2010). Community case management of fever due to malaria and pneumonia in children under five in Zambia: a cluster randomized controlled trial.. PLoS Med.

[16] Druetz T, Siekmans K, Goossens S, Ridde V, Haddad S (2013). The community case management of pneumonia in Africa: a review of the evidence.. Health Policy Plan.

[17] Gilroy KE, Callaghan J, Cardemil C, Nsona H, Amouzou A, Mtimuni A (2013). Quality of sick child care delivered by community–based health workers in Malawi.. Health Policy Plan.

[18] Miller NP, Amouzou A, Tafesse M, Hazel E, Legasse H, Degefie T (2014). Integrated community case management of childhood illness in Ethiopia: Implementation strength and quality of care.. Am J Trop Med Hyg.

[19] Bryce J, Victora CG, Habicht JP, Vaughan JP, Black RE (2004). The multicountry evaluation of integrated management of childhood illness strategy: lessons for the evaluation of public health interventions.. Am J Public Health.

[20] Bryce J, Victora C (2005). Ten methodological lessons from the multi–country evaluation of the integrated management of childhood illness.. Health Policy Plan.

[21] Armstrong Schellenberg JR, Adam T, Mshinda H, Masanja H, Kabadi G, Mukasa O (2004). Effectiveness and cost of facility–based Integrated Management of Childhood Illness (IMCI) in Tanzania.. Lancet.

[22] Arifeen SE, Hoque DME, Akter T, Rahman M, Hoque ME, Begum K (2009). Effect of the Integrated Management of Childhood Illness strategy on childhood mortality and nutrition in a rural area in Bangladesh: a cluster randomised trial.. Lancet.

[23] Victora CG, Habicht JP, Bryce J (2004). Evidence–Based Public Health: Moving Beyond Randomized Trials.. Am J Public Health.

[24] Victora CG, Black RE, Boerma T, Bryce J (2011). Measuring impact in the Millennium Development Goal era and beyond: a new approach to large–scale effectiveness evaluations.. Lancet.

[25] Chinbuah MA, Kager PA, Abbey M, Gyapong M, Awini E, Nonvigon J (2012). Impact of community management of fever (using antimalarials with or without antibiotics) on childhood mortality: a cluster–randomized controlled trial in Ghana.. Am J Trop Med Hyg.

[26] Hill K (1991). Approaches to the measurement of childhood mortality: a comparative review.. Popul Index.

[27] Amouzou A, Banda B, Kachaka W, Joos O, Kanyuka M, Hill K (2014). Monitoring child mortality through community health worker reporting of births and deaths in Malawi: validation against a household mortality survey.. PLoS ONE.

[28] Diaz T, Guenther T, Oliphant NP, Muńiz M (2014). iCCM Symposium impact outcome evaluation thematic group. A proposed model to conduct process and outcome evaluations and implementation research of child health programs in Africa using integrated community case management as an example.. J Glob Health.

[29] Guenther T, Barberá LZ, Oliphant N, Dale M, Raharison S, Miller L (2014). Routine monitoring systems for integrated community case management programs: lessons from 18 countries in sub–Saharan Africa.. J Glob Health.

[30] Hazir T, Nisar YB, Abbasi S, Ashraf YP, Khurshid J, Tariq P (2011). Comparison of oral amoxicillin with placebo for the treatment of world health organization–defined nonsevere pneumonia in children aged 2–59 months: a multicenter, double–blind, randomized, placebo–controlled trial in Pakistan.. Clin Infect Dis.

